# Relationships between Skin Carotenoid Levels and Metabolic Syndrome

**DOI:** 10.3390/antiox11010014

**Published:** 2021-12-22

**Authors:** Yuji Takayanagi, Akira Obana, Shigeki Muto, Ryo Asaoka, Masaki Tanito, Igor V. Ermakov, Paul S. Bernstein, Werner Gellermann

**Affiliations:** 1 Department of Ophthalmology, Faculty of Medicine, Shimane University, Izumo 693-8501, Japan; y.takayanagi1008@gmail.com (Y.T.); mtanito@med.shimane-u.ac.jp (M.T.); 2 Department of Ophthalmology, Seirei Hamamatsu General Hospital, Hamamatsu 430-8558, Japan; ryoasa0120@googlemail.com; 3 Department of Medical Spectroscopy, Institute for Medical Photonics Research, Preeminent Medical Photonics Education & Research Center, Hamamatsu University School of Medicine, Hamamatsu 431-3192, Japan; 4 Seirei Center for Health Promotion and Prevention Medicine, Seirei Social Welfare Community, Hamamatsu 430-0906, Japan; smuto@sis.seirei.or.jp; 5 Longevity Link Corporation, Salt Lake City, UT 84108, USA; iv.ermakov@outlook.com (I.V.E.); wbgellermann@gmail.com (W.G.); 6 Department of Ophthalmology and Visual Sciences, Moran Eye Center, University of Utah School of Medicine, Salt Lake City, UT 84132, USA; paul.bernstein@hsc.utah.edu

**Keywords:** metabolic syndrome, health examination population, skin carotenoids, oxidative stress, Veggie Meter

## Abstract

Carotenoids have potential antioxidant and anti-inflammatory effects; their protective roles are of particular interest in the pathogenesis of metabolic syndrome (MetS). The reflection spectroscopy method has been recently developed to noninvasively measure skin carotenoid (SC) levels, which highly correlates with serum concentration of carotenoids. The relationship between SC levels and metabolic syndrome has been investigated. We aimed to identify the differences in patient characteristics and SC levels between participants with and without MetS in a large health examination population. In addition, the relationships between SC levels and various clinical parameters related to MetS were investigated. SC levels were measured using a reflection spectroscopy. A total of 1812 Japanese participants (859 male, 953 female; mean age ± standard deviation (SD), 57.8 ± 11.0 years) comprised the study population, i.e., participants with MetS (*n* = 151) and those without MetS (*n* = 1661). Multivariate logistic regression analysis was performed to identify variables associated with MetS. Compared to controls (377.3 ± 122.8), SC indices were significantly lower in patients with MetS (340.7 ± 112.5, *p* = 0.0004). Multivariate models also suggested that lower SC was significantly associated with MetS after adjustment for age, sex, smoking habit, and other potential risk factors for MetS. Furthermore, male gender (*p* < 0.0001), smoking habit (*p* < 0.0001) and worse lipid profiles (i.e., serum triglyceride (*r* = −0.1039, *p* < 0.0001), high-density lipoprotein (*r* = 0.1259, *p* < 0.0001), and usage of hypolipidemic agents (*p* = 0.0340)) were significantly associated with lower SC levels. The current study indicated that lower SC levels were significantly associated with MetS. This study highlights the antioxidant capacity of carotenoids in patients with MetS and the clinical utility of non-invasive and cost-effective SC measurement to detect participants who are at risk of developing MetS in a large population.

## 1. Introduction

Metabolic syndrome (MetS) is characterized by the clustering of metabolic abnormalities that include anthropometric and physiological parameters defined by several criteria [1,2]. Existing data suggest that its prevalence has been on the rise for decades [3,4]. Oxidative stress and chronic inflammation have been suggested to contribute to MetS pathogenesis; conversely, MetS accelerates oxidation and inflammation in various organs and tissues that causes various diseases, although the exact mechanisms underlying the relationship among them remain unclear.

Carotenoids, organic pigments produced by plants and algae, are one of the most major antioxidants that quench free radicals and inhibit lipid peroxidation [5,6]. Since carotenoids have potential antioxidant and anti-inflammatory effects, their protective roles are of particular interest in the pathogenesis of MetS [7,8]. However, the association between dietary carotenoid intake and the prevalence of MetS is still controversial. Some reports have shown a significant association between carotenoid consumption and MetS and a positive effect of carotenoid intake to prevent MetS; others have failed to reveal a significant association [9]. The insufficient evidence between dietary consumption and MetS is considered to be due to the bioavailability of carotenoids. Several factors affect the bioavailability of carotenoids [10], and there are individual differences in the absorption of carotenoids from the diet and supplements. In contrast, the significant association between serum concentrations of carotenoids and MetS has been admitted previously [2,7,11,12,13,14,15]. High serum concentrations of carotenoids work negatively for MetS development.

Recently, the reflection spectroscopy (RS) method has been developed to noninvasively measure SC levels used for scientific research and in medical, industrial, and nutritional fields [16,17,18,19]. Carotenoids accumulate in the epidermis by diffusion from the subcutaneous fat, blood, and lymph flows and secretion on the skin surface from the sweat glands and sebaceous glands [20]. Plasma or serum carotenoids reflect recent dietary intake for up to 2 weeks; however, the deposition of carotenoids in the skin increases their longevity for up to 4 weeks after intake [17]. SC levels correlated with serum concentrations of total carotenoids [21,22,23,24,25,26,27,28]. Conrady et al. summarized the results and showed high correlation coefficients between SC levels and total carotenoid concentrations in serum [29]. Similar to the relationship between the serum concentration and dietary consumption, the relationship between SC levels and dietary consumption is moderate, due to bioavailability. Measuring carotenoid concentrations in plasma or serum is a gold standard of carotenoid investigation; however, it is mildly invasive and time consuming. In contrast, the simple nature of the RS method is a useful alternative and allows us to investigate SC levels in a large population. However, few studies have examined the correlations between SC levels and MetS.

In the present study, we aimed to compare the differences in patient characteristics and SC levels between participants with and without MetS in a health examination population with a large sample size and to explore the antioxidant capacity of carotenoids in relation to the MetS. In addition, the relationships between SC levels and various clinical parameters related to MetS, such as lipid profiles, were investigated.

## 2. Methods

### 2.1. Participants

The current study adhered to the tenets of the Declaration of Helsinki. The Institutional Review Boards of Seirei Hamamatsu General Hospital and Seirei Center for Health Promotion and Prevention Medicine approved the research (IRB No. 3030, 31-02). All participants provided written informed consent for inclusion in the study. All participants’ information was anonymized. We included participants who consented to this study and received health examinations, including SC measurement in Seirei Center for Health Promotion and Prevention Medicine from September 2019 to July 2020. Participants were 1812 people with a mean age of 57.8 ± 11.0 (standard deviation, SD) years. There were no exclusion criteria in this study. Males accounted for 859 participants (47.4%), and females for 953 (52.6%). Participants with MetS (*n* = 151) comprised of 120 males and 31 females; those without MetS (*n* = 1661) comprised of 739 males and 922 females. Supplementary Figure S1 provides a flow diagram describing the selection of the population for analysis. The participants were interviewed about their smoking habits and usage of antihypertensive agents, hypolipidemic agents, oral diabetes drugs, and insulin. The participants also underwent physiological examinations, i.e., body mass index (BMI), body fat percentage, waist circumference (WC), systolic and diastolic blood pressures, heart rate, measurement of skin carotenoid levels, and blood tests, including white blood count (WBC), hematocrit (Hct), aspartate aminotransferase (AST), alanine aminotransferase (ALT), γ-glutamyl transpeptidase (γ-GTP), alkaline phosphatase (ALP), lactate dehydrogenase (LDH), cholinesterase (ChE), zinc sulfate turbidity test (ZTT), total bilirubin (Bil), total protein (TP), albumin, blood urea nitrogen (BUN), creatinine (Cre), estimated glomerular filtration rate (eGFR), uric acid (UA), total cholesterol, fasting triglyceride (TG), high-density lipoprotein (HDL), low-density lipoprotein (LDL), non-high-density lipoprotein (non-HDL), fasting blood glucose, hemoglobin A1c (HbA1c), amylase, lipase, and C-reactive protein. We measured HDL, LDL, and non-HDL cholesterol as cholesterol fractionation. MetS was diagnosed according to the Examination Committee of Criteria defined by the Japanese Society for the Study of Obesity (i.e., WC > 85 cm in male, >90 cm in female plus two or more of the following were present: HDL-C < 40 mg/dL, TG> 150 mg/dL, or use of antilipidemic agents, and/or systolic blood pressure >130 mmHg, diastolic blood pressure >85 mmHg, or use of antihypertensive agents, and/or fasting plasma glucose >110 mg/dL or antidiabetic agents) [30].

### 2.2. Measurement of Skin Carotenoid Levels

SC levels were measured using pressure-mediated RS (Veggie Meter^®^, Longevity Link Corporation, Salt Lake City, UT, USA). The basics of Veggie Meter have been described elsewhere [16]. It can spare the influence of blood perfusion by the pressure on the finger-tip and measure SC levels with little influence of melamine pigment [18]. We confirmed the usefulness of Veggie Meter in Japanese in our previous study [19].Measurements were performed following the instructions of the device manufacturer. Calibration was performed with the manufacturer-provided reference materials prior to daily skin measurements twice a day (before the morning session and before the afternoon session). In the measurement of SC, participants inserted the left middle finger into the device’s finger cradle. The SC index was determined as the average of three consecutive measurements in 1796 participants and by single measurements in 16 participants. 

### 2.3. Statistical Analysis

For comparisons between the two groups, the differences in continuous data were investigated using the unpaired student’s *t*-test, and the differences in categorical data were analyzed using the Fisher’s exact probability test. The correlations between skin carotenoids index and clinical parameters, i.e., age, waist circumference, systolic blood pressure, heart rate, and various laboratory data, were assessed using Pearson’s correlation test, for which *p* values of 0.05 were considered statistically significant. To determine independent factors associated with MetS, we also performed multivariate logistic regression analysis between the presence of MetS and various covariates (age, sex, the presence of smoking habit, WBC, UA, LDL, and skin carotenoid). All statistical analyses were calculated using JMP Pro statistical software version 14.2 (SAS Institute, Inc., Cary, NC, USA). All reported *p* values are two-sided. The data are expressed as the means ± SD for continuous variables and in numbers and percentage for categorical variables. 

## 3. Results

The subject data, including age, sex, presence of smoking habit, antihypertensive agents use, hypolipidemic agents use, oral diabetes drugs or insulin usage, obesity parameters such as BMI, body fat percentage and waist circumference, systolic and diastolic blood pressures, heart rate, and SC index, are shown in Table 1. Compared to the non-MetS group, higher mean age, large percentage of male gender, and worse obesity parameters, including BMI and body fat percentage, were observed in the MetS group. MetS group also showed higher values in parameters included as a definition of MetS, such as frequency of the usage of antihypertensive agents, hypolipidemic agents, and oral diabetes drugs/regular insulin, higher waist circumference, and higher systolic and diastolic blood pressures. SC indices were significantly lower in patients with MetS than those with non-MetS (*p* = 0.0004). The subject data stratified by age quartiles is shown in Supplementary Table S1.

Table 2 summarizes the comparison of various laboratory data between the two groups. Worse blood glucose control, i.e., fasting blood TG, fasting BS, and HbA1c and worse lipid profile, i.e., lower HDL and higher TC, and non-HDL were observed in MetS group than non-MetS group. Higher RBC counts, WBC counts, Hb, Hct, liver function tests, i.e., AST, ALT, γ-GTP, ALP, LDH, ChE, Bil, TP, BUN, creatinine, eGFR, and UA also were found in patients with MetS than in those without MetS. The other parameters, including ZTT, Alb, LDL, amylase, lipase, and CRP, did not differ significantly.

Table 3 demonstrates multiple logistic regression models of factors potentially associated with MetS, which included age, sex, smoking habit, WBC, UA, LDL, and SC levels. The model indicated that age, male gender, higher WBC counts, higher UA, and lower SC levels were independent variables that were significantly associated with MetS. 

The clinical parameters that were associated with SC levels were shown in Table 4 and Table 5. Among continuous variables (Table 4), positive correlations with SC levels were admitted in age, LDH, ChE, Bil, BUN, T-Cho, HDL, amylase, and lipase and negative correlations were found for waist circumference, RBC, WBC, Hct, ALT, γ-GTP, ChE, Cre, UA, TG, Fasting BS. Among categorical variables (Table 5), male gender, presence of smoking habit, and usage of hypolipidemic agents were correlated with significantly lower SC levels.

## 4. Discussion

This study was designed to investigate the association between SC levels and MetS, and to elucidate the potential role of carotenoids in patients with MetS. Overall, the current study suggested two important clinical findings. First, the SC levels were significantly lower in patients with MetS than non-MetS subjects, and lower SC levels were independently associated with MetS. Second, male gender, smoking habit and worse lipid profiles were significantly associated with lower SC levels. 

The present study revealed significantly lower SC levels in patients with MetS than those without MetS and the multivariate logistic regression analysis showed that lower SC levels were independently associated with MetS. Several previous studies have reported the association between serum carotenoid concentration and MetS [2,7,11,12,13,14,15]. Since SC levels correlated with serum concentrations of total carotenoids [21,22,23,24,25,26,27,28], the present result was well acceptable. An explanation for this relationship is that serum carotenoids play an important role in the chronic inflammation co-occurring with oxidative stress [31]. Oxidative stress is evidently associated with MetS, in which irreversible accumulation of oxidation products in proteins, lipids, and glucoses, induce the impairment of intracellular redox signaling pathways and detrimentally affect vascular remodeling and insulin resistance [32]. Although increased oxidative stress has been implicated with the pathogenesis of MetS, carotenoids can act as direct antioxidants, quenching singlet oxygen and reducing the formation of lipid peroxides [33], which are positively correlated with insulin resistance [34]. Indeed, negative correlations between serum carotenoids and markers of oxidative stress have been reported [35], especially in studies with patients in poor health, such as people with diabetes and MetS. Thus, it is biologically plausible that carotenoids contribute to the pathogenesis of MetS via oxidative stress-induced signaling pathways and low levels of carotenoid in the human body is speculated to be a risk factor of developing MetS. 

The non-invasive measurement of SC levels by RS could be used as a reasonable and reliable examination to find patients with a risk of MetS. Since carotenoids cannot be synthesized in the human body, the lower SC levels indicates lower dietary carotenoid intake and may reflect worse dietary patterns. Although the previous study demonstrated the discrepancy of protective effects between dietary intake of carotenoids and serum carotenoids levels [36], the bioavailability of carotenoids may explain this discrepancy. Several factors which may affect the carotenoid bioavailability, including carotenoid species, vitamin status, and genetic factors, were reported [10]. Therefore, the serum concentration of carotenoids and SC levels have more direct association with MetS than dietary consumption.

In the current study, SC levels were negatively correlated with male gender and smoking habit. Previous reports have shown the association between SC levels and both male gender and smoking habit [7,11], which is in line with the present results. Several studies have reported that smoking was associated with increased risk of MetS [37,38]. Antioxidants, including serum carotenoids, may have a key role for the prevention of MetS, especially in smokers who are exposed to high oxidative stress. Other previous reports have demonstrated the relationship between serum carotenoids and dietary intake of vegetables and fruits; moreover, women consume higher amounts of carotenoids than men [39], which might explain the gender discrepancy of SC levels. In the present study, SC levels of participants taking hypolipidemic agents were significantly higher than those of participants without taking them. The definite mechanisms of this difference were unclear. The alteration of lipid metabolisms by hypolipidemic agents and the change of mind in participants who received nutrition education before taking such agents may have influenced the results. 

It is also important to note that a positive correlation between SC levels and HDL was observed in this study. Since lutein and zeaxanthin are primarily transported in plasma by HDL [40,41], as decrease in HDL is associated with a tissue decrease in xanthophylls [42]. Therefore, the correlation between SC levels and HDL was biologically explainable. Furthermore, Xanthophylls such as lutein and zeaxanthin are major carotenoids that have important bioactivity in humans because of their protective effects against oxidative stress. In fact, one systematic review showed that higher lutein serum concentration or intake was associated with a lower risk of MetS, as well as coronary heart disease and stroke [43]. The correlation between SC levels and HDL in the current study strongly supported our hypothesis that a decrease in xanthophylls induced by decreased HDL might exert an adverse influence on the pathogenesis of MetS. 

Lastly, there were several limitations to the present study that are noteworthy, as they may affect the generalization of our findings. First, our study has the same limitations of any cross-sectional study in being neither controlled nor randomized. Second, the senile aged and relatively healthy population could limit the generalization of our results. The present participants received a paid health examination at their own will. Since most participants were highly health conscious, relatively few patients with MetS were included in this study. This warrants further research, including in general populations, such as health examinations in the workplace or community. Third, no detailed dietary questionnaire was obtained in this examination program. Fourth, caution should be taken with the interpretation of the lipid profiles in this study due to the usage of hypolipidemic agents, which can create potential selection bias. Finally, the present study evaluated total carotenoid levels, including xanthophyll carotenoids and carotenes in the skin. The distribution of carotenoids in human tissues varies according to the carotenoid species and each type has specific physiological effects. This can limit the usage of SC measurement and affect the interpretations of the result in this study. Despite these limitations, our study has many strengths, including a large sample size of individuals and comprehensive assessments of patients’ clinical characteristics, physical examinations, and laboratory data. There have been no studies that investigated in detail the association of SC levels and comprehensive laboratory data. The present report provided the first results in a large population.

## 5. Conclusions

In conclusion, the current study suggested that lower SC levels were observed in patients with MetS compared to those without MetS. Male gender, smoking habit and worse lipid profiles were significantly correlated with lower SC levels. The SC measurement, rather than serum carotenoid, is a non-invasive, cost-effective and highly reliable method in clinical settings. Measurement of SC levels to evaluate carotenoid status might be useful to detect the occurrence and development of MetS. Our findings warrant further research to explore the underlying mechanisms of antioxidative effects induced by carotenoids in patients with MetS and to investigate the clinical utility of this method to prevent MetS.

## 6. Patents

I.V.E. and W.G. hold patents of “Noninvasive Measurement of Carotenoids in Biological Tissue.” U.S. Patent # 8,260,402 granted 2012, Japanese Patent JP 5574246B2 granted 2014. 

## Figures and Tables

**Table 1:** Data of study subjects with and without metabolic syndrome.

	Metabolic Syndrome	Non-Metabolic Syndrome	*p*-Value
N	151	1661	
Age (years)			
Mean ± SD	62.0 ± 8.5	57.4 ± 11.1	<0.0001 **
range	40.0, 80.0	22.0, 90.0	
Sex			
Men, *n* (%)	120 (79.5)	739 (44.5)	<0.0001 **
Women, *n* (%)	31 (20.5)	922 (55.5)	
Smoking habit			
Yes, *n* (%)	11 (7.3)	60 (3.6)	0.0438 *
No, *n* (%)	140 (92.7)	1601 (96.4)	
Antihypertensive agents			
Yes, *n* (%)	101 (66.9)	233 (14.0)	<0.0001 **
No, *n* (%)	50 (33.1)	1428 (86.0)	
Hypolipidemic agents			
Yes, *n* (%)	83 (55.0)	219 (13.2)	<0.0001 **
No, *n* (%)	68 (45.0)	1442 (86.8)	
Oral diabetes drugs/Insulin usage			
Yes, *n* (%)	21 (13.9)	47 (2.8)	<0.0001 **
No, *n* (%)	130 (86.1)	1614 (97.2)	
BMI (kg/m^2^)			
Mean ± SD	26.8 ± 3.3	22.2 ± 3.0	<0.0001 **
range	22.0, 42.0	14.0, 44.0	
Body fat percentage (%)			
Mean ± SD	28.8 ± 7.0	24.1 ± 6.5	<0.0001 **
range	17.0, 50.0	8.0, 58.0	
Waist circumference (cm)			
Mean ± SD	94.3 ± 7.1	80.0 ± 8.4	<0.0001 **
range	85.0, 132.0	59.0, 120.0	
Systolic Blood pressure (mmHg)			
Mean ± SD	127.7 ± 12.4	116.3 ± 15.1	<0.0001 **
range	90.0, 160.0	80.0, 194.0	
Diastolic blood pressure (mmHg)			
Mean ± SD	78.2 ± 9.8	71.5 ± 9.8	<0.0001 **
range	56.0, 110.0	42.0, 110.0	
Heart rate (bpm)			
Mean ± SD	64.0 ± 10.2	62.7 ± 9.7	0.1219
range	41.0, 92.0	38.0, 125.0	
Skin carotenoid			
Mean ± SD	340.7 ± 112.5	377.3 ± 122.8	0.0004 **
range	167.0, 772.0	83.0, 974.0	

Comparison between metabolic syndrome and non-metabolic syndrome groups by using unpaired student’s t test for continuous data and by using Fisher’s exact probability test for categorical data. The * and ** correspond to the significance levels at 5% (*p* < 0.05) and 1% (*p* < 0.01), respectively. N, number of participants; SD, standard deviation; bpm, beats per minute.

**Table 2:** Summary of laboratory data of study subjects with and without metabolic syndrome.

	Metabolic Syndrome		Non-Metabolic Syndrome		
	Mean ± SD	Range	Mean ± SD	Range	*p*-Value
*N*	151		1661		
RBC (10^4^/μL)	479.6 ± 40.8	378, 598	453.8 ± 42.6	272, 631	<0.0001 **
WBC (/μL)	5797.2 ± 1394.2	2880, 11,310	4934.4 ± 1239.8	2280, 13,210	<0.0001 **
Hb (g/dL)	14.9 ± 1.2	11.5, 17.5	13.9 ± 1.4	5.3, 18.4	<0.0001 **
Hct (%)	43.8 ± 3.3	35.0, 51.8	41.5 ± 3.7	20.4, 53.9	<0.0001 **
AST (IU/L)	27.4 ± 14.9	13, 146	21.3 ± 11.0	7, 388	<0.0001 **
ALT (IU/L)	34.7 ± 26.2	10, 191	19.7 ± 12.4	6, 232	<0.0001 **
γ-GTP (IU/L)	50.8 ± 48.7	8, 345	28.1 ± 29.0	6, 450	<0.0001 **
ALP (IU/L)	220.2 ± 58.7	109, 444	204.4 ± 58.2	65, 638	0.0015 **
LDH (IU/L)	188.7 ± 33.0	126, 349	179.5 ± 37.0	69, 1069	0.0032 *
ChE (IU/L)	373.2 ± 60.0	230, 549	333.2 ± 71.7	134, 1029	<0.0001 **
ZTT (KU)	7.6 ± 3.3	1, 16	8.0 ± 3.4	1, 28	0.2040
Bil (mg/dL)	0.99 ± 0.4	0.4, 3.4	0.93 ± 0.34	0.3, 3.5	0.0235 *
TP (g/dL)	7.3 ± 0.4	6.4, 8.6	7.2 ± 0.4	6.0, 8.7	0.0009 **
Albumin (g/dL)	4.3 ± 0.2	3.7, 5.0	4.3 ± 0.2	3.2, 5.3	0.2289
BUN (mg/dL)	15.8 ± 4.3	8, 30	14.6 ± 3.6	6, 33	<0.0001 **
Cre (mg/dL)	0.9 ± 0.3	0.52, 2.34	0.7 ± 0.2	0.33, 1.48	<0.0001 **
eGFR (mL/min/1.73 m^3^)	65.8 ± 14.3	24.2, 98.7	73.8 ± 13.6	30.9, 145.5	<0.0001 **
UA (mg/dL)	6.0 ± 1.3	3.0, 9.3	5.2 ± 1.2	0.5, 9.3	<0.0001 **
T-Chol (mg/dL)	206.3 ± 37.0	116, 324	214.6 ± 33.2	87, 327	0.0034 **
Fasting TG (mg/dL)	162.2 ± 146.3	43, 1670	91.9 ± 58.9	19, 1037	<0.0001 **
HDL (mg/dL)	57.3 ± 13.1	29, 107	73.1 ± 18.9	30, 158	<0.0001 **
LDL (mg/dL)	124.2 ± 30.7	43, 225	127.0 ± 29.3	35, 239	0.2559
non-HDL (mg/dL)	149.0 ± 36.5	78, 259	141.6 ± 32.1	38, 267	0.0074 **
Fasting BG (mg/dL)	112.4 ± 20.3	81, 217	94.3 ± 11.7	57, 184	<0.0001 **
HbA1c (%)	6.2 ± 0.7	5.1, 8.2	8.7 ± 0.4	4.7, 8.7	<0.0001 **
Amylase (IU/L)	83.6 ± 33.6	30, 218	85.0 ± 28.3	28, 318	0.6365
Lipase (IU/L)	32.5 ± 11.5	8, 85	34.0 ± 12.2	8.0, 249.0	0.1376
CRP (mg/dL)	0.16 ± 0.20	0.01, 1.27	0.10 ± 0.51	0.0, 17.4	0.1701

Comparison between metabolic syndrome and non-metabolic syndrome groups by using unpaired student’s t test for continuous data. The * and ** correspond to the significance levels at 5% (*p* < 0.05) and 1% (*p* < 0.01), respectively. *N*, number of participants; SD, standard deviation; IU, international unit; RBC, red blood cell; WBC white blood count; Hb, hemoglobin; Hct, hematocrit; AST, aspartate aminotransferase; ALT, alanine aminotransferase; γ-GTP, γ-glutamyl transpeptidase; ALP, alkaline phosphatase; LDH, lactate dehydrogenase; ChE, cholinesterase; ZTT, zinc sulfate turbidity test; KU, kunkel unit; Bil, total bilirubin; TP, total protein; BUN, blood urea nitrogen; Cre, creatinine; eGFR, estimated glomerular filtration rate; UA, uric acid; T-Chol, total cholesterol; TG, triglyceride; HDL, high-density lipoprotein; LDL, low-density lipoprotein; non-HDL, non-high-density lipoprotein; BG, blood glucose; HbA1c, hemoglobin A1c; CRP, C-reactive protein.

**Table 3:** Multiple regression analysis for risk factors of metabolic syndrome.

	OR	Inverse of OR	95%CI	*p*-Value
Entire model				<0.0001 **
Age (/years)	1.0550	0.9478	1.0356, 1.0749	<0.0001 **
Sex (male/female)	2.9514	0.3388	1.8572, 4.6902	<0.0001 **
Smoking habit (yes/no)	1.1390	0.8779	0.5497, 2.3602	0.7262
WBC (/units)	1.0004	0.9996	1.0000, 1.0003	<0.0001 **
UA (/units)	1.2275	0.8147	1.0443, 1.4428	0.0129 *
LDL (/units)	0.9976	1.0024	0.9915, 1.0037	0.4455
Skin carotenoid (/units)	0.9973	1.0026	0.9973, 0.9956	0.0023 **

Multivariate logistic regression analysis was performed with the following factors: age, sex, the presence of smoking habit, WBC, UA and skin carotenoid. *p* values were calculated using the likelihood ratio test. Odds ratios for continuous variables are expressed as the odds ratio associated with a one-unit increase. The * and ** correspond to the significance levels at 5% (*p* < 0.05) and 1% (*p* < 0.01), respectively. OR, odds ratio; CI, confidence interval; WBC, white blood cell; UA, uric acid.

**Table 4:** Associations among skin carotenoid levels and various continuous parameters.

	*r*	Lower 95% CI	Upper 95% CI	*p*-Value
Age	0.2212	0.1770	0.2646	<0.0001 **
Waist circumference	−0.1957	−0.2396	−0.1510	<0.0001 **
Systolic Blood pressure	−0.0271	−0.0730	0.0190	0.2491
Heart rate	0.0254	−0.0207	0.0714	0.2801
RBC	−0.0872	−0.1327	−0.0413	0.0002 **
WBC	−0.1003	−0.1456	−0.0545	<0.0001 **
Hct	−0.0676	−0.1133	−0.0216	0.0040 **
AST	−0.0149	−0.0609	0.0312	0.5272
ALT	−0.1007	−0.1461	−0.0549	<0.0001 **
γ-GTP	−0.1438	−0.1886	−0.0984	<0.0001 **
ALP	0.0086	−0.0375	0.0546	0.715
LDH	0.0804	0.0345	0.1260	0.0006 **
ChE	−0.0841	−0.1297	−0.0382	0.0003 **
ZTT	−0.0222	−0.0784	0.0341	0.4392
Bil	0.1198	0.0741	0.1650	<0.0001 **
TP	0.0393	−0.0067	0.0852	0.0941
Albumin	0.0236	−0.0225	0.0695	0.3161
BUN	0.1120	0.0663	0.1572	<0.0001 **
Cre	−0.0884	−0.1339	−0.0425	0.0002 **
UA	−0.1296	−0.1747	−0.0841	<0.0001 **
T-Chol	0.0605	0.0145	0.1063	0.0100 **
TG	−0.1039	−0.1493	−0.0581	<0.0001 **
HDL	0.1259	0.0803	0.1710	<0.0001 **
LDL	0.0237	−0.0223	0.0697	0.3128
non-HDL	−0.0108	−0.0568	0.0353	0.6460
Fasting BG	−0.0491	−0.0950	−0.0030	0.0367 *
HbA1c	0.0344	−0.0117	0.0803	0.1431
Amylase	0.1348	0.0792	0.1896	<0.0001 **
Lipase	0.1593	0.1140	0.2039	<0.0001 **
CRP	−0.0243	−0.0703	0.0218	0.3009

The correlation coefficient (r) and *p* values for each pair of groups, calculated by using Pearson’s correlation test. The * and ** correspond to the significance levels at 5% (*p* < 0.05) and 1% (*p* < 0.01), respectively. CI, confidence interval; RBC, red blood cell; WBC white blood count; Hct, hematocrit; AST, aspartate aminotransferase; ALT, alanine aminotransferase; γ-GTP, γ-glutamyl transpeptidase; ALP, alkaline phosphatase; LDH, lactate dehydrogenase; ChE, cholinesterase; ZTT, zinc sulfate turbidity test; Bil, total bilirubin; TP, total protein; BUN, blood urea nitrogen; Cre, creatinine; UA, uric acid; T-Chol, total cholesterol; TG, triglyceride; HDL, high-density lipoprotein; LDL, low-density lipoprotein; non-HDL, non-high-density lipoprotein; BG, blood glucose; HbA1c, hemoglobin A1c; CRP, C-reactive protein.

**Table 5:** Associations among skin carotenoid levels and various categorical parameters.

	*N*	Mean ± SD	Range	*p*-Value
Sex				
Male	859	354.6 ± 118.0	132.0, 925.0	<0.0001 **
Female	953	391.9 ± 123.5	83.0, 974.0	
Smoking habit				
Yes	71	287.9 ± 77.4	175.0, 650.0	<0.0001 **
No	1741	377.7 ± 122.5	83.0, 974.0	
Antihypertensive agents				
Yes	334	377.4 ± 128.7	137.0, 847.0	0.5991
No	1478	373.5 ± 120.9	83.0, 974.0	
Hypolipidemic agents				
Yes	302	387.9 ± 123.8	137.0, 834.0	0.0340 *
No	1510	371.5 ± 121.3	83.0, 974.0	
Insulin usage				
Yes	68	367.0 ± 134.1	132.0, 784.0	0.6177
No	1744	374.5 ± 121.9	83.0, 974.0	

The *p* values are calculated by using the unpaired student’s *t*-test between groups. The * and ** correspond to the significance levels at 5% (*p* < 0.05) and 1% (*p* < 0.01), respectively. N, number; SD, standard deviation.

## Data Availability

The data presented in this study are available in this manuscript.

## Supplementary Materials

The following are available online at https://www.mdpi.com/article/10.3390/antiox11010014/s1, Figure S1: Flow diagram of selecting the population for analysis from the Japanese health examination cohort. There were no exclusion criteria in this study. Table S1: Demographic data of study participants stratified by age quartiles.

## Abbreviations

MetS	metabolic syndrome
SC	skin carotenoid
RS	reflection spectroscopy
BMI	body mass index
WC	waist circumference
WBC	white blood count
Hct	hematocrit
AST	aspartate aminotransferase
ALT	alanine aminotransferase
γ-GTP	γ-glutamyl transpeptidase
ALP	alkaline phosphatase
LDH	lactate dehydrogenase
ChE	cholinesterase
ZTT	zinc sulfate turbidity test
Bil	total bilirubin
TP	total protein
BUN	blood urea nitrogen
Cre	creatinine
eGFR	estimated glomerular filtration rate
UA	uric acid
TG	fasting triglyceride
HDL	high-density lipoprotein
LDL	low-density lipoprotein
non-HDL	non-high-density lipoprotein
HbA1c	hemoglobin A1c
