# Supplementary material for: Relationships between Skin Carotenoid Levels and Metabolic Syndrome
Source: Antioxidants (Basel). 2021 Dec 22;11(1):14. doi: 10.3390/antiox11010014 (PMC8772725; doi:10.3390/antiox11010014)
Supplement: Supplementary file 1 [file antioxidants-11-00014-s001.zip › antioxidants-1487226-supplementary.pdf]

antioxidants

MDPI

Article

Relationships between skin carotenoid levels and metabolic syndrome

Yuji Takayanagi[1], Akira Obana[2,3*], Shigeki Muto[4], Ryo Asaoka[2], Masaki Tanito[1], Igor V Ermakov[5], Paul S Bernstein[6], Werner Gellermann[5]

Supplementary Table 1. Demographic data of study participants stratified by age quartiles

	Age ≤ 50			50 < Age ≤ 59			59 < Age ≤ 66			66 < Age		
	Non-MetS	MetS	*P*-value	Non-MetS	MetS	*P*-value	Non-MetS	MetS	*P*-value	Non-MetS	MetS	*P*-value
N	453	16		462	39		375	49		371	47	
Age (years)												
Mean ± SD	43.4 ± 5.8	46.8 ± 3.7	0.0200*	55.1 ± 2.6	55.8 ± 2.6	0.0903	62.8 ± 2.1	62.7 ± 1.8	0.6791	71.8 ± 4.0	71.6 ± 3.7	0.8059
range	22, 50	40, 50		51, 59	51, 59		60, 66	60, 66		67, 90	67, 80	
Sex												
Men, n (%)	199 (43.9)	14 (87.5)	0.0006**	205 (44.4)	31 (79.5)	<0.0001**	163 (43.5)	35 (71.4)	0.0002**	172 (46.4)	40 (85.1)	<0.0001**
Women, n (%)	254 (56.1)	2 (12.5)		257 (55.6)	8 (20.5)		212 (56.5)	14 (28.6)		199 (53.6)	7 (14.9)	
Smoking habit												
Yes, n (%)	31 (6.8)	4 (25.0)	0.0249*	12 (2.6)	2 (5.1)	0.2985	13 (3.5)	4 (8.2)	0.1206	4 (1.1)	1 (2.1)	0.4509
No, n (%)	422 (93.2)	12 (75.0)		450 (97.4)	37 (94.9)		362 (96.5)	45 (91.8)		367 (98.9)	46 (97.9)	
Antihypertensive agents												
Yes, n (%)	15 (3.3)	6 (37.5)	<0.0001**	39 (8.4)	21 (53.9)	<0.0001**	67 (17.9)	36 (73.5)	<0.0001**	112 (30.2)	38 (80.9)	<0.0001**

No, n (%)	438 (96.7)	10 (62.5)		423 (91.6)	18 (46.2)		308 (82.1)	13 (26.5)		259 (69.8)	9 (19.1)	
Antihyperlipidemic agents												
Yes, n (%)	8 (1.8)	5 (31.3)	<0.0001**	43 (9.3)	22 (56.4)	<0.0001**	62 (16.5)	25 (51.0)	<0.0001**	106 (28.6)	31 (66.0)	<0.0001**
No, n (%)	445 (98.2)	11 (68.8)		419 (90.7)	17 (43.6)		313 (83.5)	24 (49.0)		265 (71.4)	16 (34.0)	
Insulin usage												
Yes, n (%)	3 (0.7)	2 (12.5)	0.0103*	6 (1.3)	5 (12.8)	0.0007**	15 (4.0)	9 (18.4)	0.0006**	23 (6.2)	5 (10.6)	0.2261
No, n (%)	450 (99.3)	14 (87.5)		456 (98.7)	34 (87.2)		360 (96.0)	40 (81.7)		348 (93.8)	42 (89.4)	
BMI (kg/m^2)												
Mean ± SD	22.3 ± 3.3	29.5 ± 2.8	<0.0001**	22.1 ± 3.0	27.6 ± 3.6	<0.0001**	22.3 ± 3.0	26.4 ± 3.2	<0.0001**	22.0 ± 2.7	25.7 ± 2.5	<0.0001**
range	16, 44	25, 36		16, 34	23, 42		14, 36	22, 35		15, 33	22, 33	
Body fat percentage (%)												
Mean ± SD	24.7 ± 6.5	31.6 ± 6.0	<0.0001**	24.1 ± 6.3	30.1 ± 6.8	<0.0001**	24.2 ± 6.7	29.2 ± 7.8	<0.0001**	23.1 ± 6.4	26.5 ± 6.0	0.0008**
range	9, 58	20, 46		11, 48	21, 50		8, 55	17, 49		8, 45	18, 45	
Waist circumference (cm)												
Mean ± SD	79.1 ± 8.7	97.1 ± 6.0	<0.0001**	79.5 ± 8.6	96.6 ± 9.0	<0.0001**	81.1 ± 8.2	93.6 ± 6.8	<0.0001**	80.4 ± 7.9	92.4 ± 5.2	<0.0001**
range	62, 120	86, 111		61, 117	86, 132		60, 111	85, 116		59, 111	85, 104	
Systolic Blood pressure (mmHg)												
Mean ± SD	109.8 ± 13.8	127.1 ± 12.1	<0.0001**	114.2 ± 14.7	128.1 ± 9.5	<0.0001**	120.1 ± 14.0	125.2 ± 13.5	0.0162*	123.0 ± 14.4	130.3 ± 13.2	0.0012**

range	84, 174	98, 144		82, 164	104, 148		80, 168	90, 160		86, 194	102, 160	
Diastolic blood pressure (mmHg)												
Mean ± SD	69.3 ± 10.0	85.6 ± 10.7	<0.0001**	72.4 ± 10.3	80.9 ± 9.3	<0.0001**	73.5 ± 8.8	76.6 ± 8.7	0.0223*	71.1 ± 9.3	75.2 ± 10.1	0.0044**
range	50, 104	70, 110		48, 110	58, 96		48, 100	60, 94		42, 100	56, 98	
Heart rate (bpm)												
Mean ± SD	62.0 ± 9.1	62.3 ± 7.1	0.9064	61.4 ± 9.5	68.9 ± 8.5	<0.0001**	64.0 ± 10.1	61.1 ± 9.3	0.0575	64.0 ± 10.0	63.5 ± 12.0	0.7827
range	38, 111	50, 77		40, 125	55, 92		40, 104	41, 86		43, 117	46, 91	
Skin carotenoid												
Mean ± SD	340 ± 101.8	315.9 ± 107.0	0.3522	366.5 ± 116.5	327.7 ± 108.6	0.0455*	380.1 ± 124.5	329.7 ± 116.5	0.0076**	433.4 ± 131.8	371.2 ± 110.6	0.0021**
range	136, 778	186, 579		83, 749	185, 745		132, 974	167, 686		137, 847	220, 772	

Comparison between metabolic syndrome and non-metabolic syndrome groups by using unpaired student t test for continuous data and by using Fisher's exact probability test for categorical data. The * and ** correspond to the significance levels at 5% (P < 0.05) and 1% (P < 0.01), respectively. MetS, Metabolic syndrome; N, number of participants; SD, standard deviation; bpm, beats per minute.
